# Time-sensitive predictors of embolism in patients with left-sided endocarditis: Cohort study

**DOI:** 10.1371/journal.pone.0215924

**Published:** 2019-04-25

**Authors:** Alvin Yang, Charlie Tan, Neill K. J. Adhikari, Nick Daneman, Ruxandra Pinto, Bennett K. M. Haynen, Gideon Cohen, Mark S. Hansen

**Affiliations:** 1 Faculty of Medicine, University of Toronto, Toronto, Ontario, Canada; 2 Sunnybrook Research Institute, Sunnybrook Health Sciences Centre, Toronto, Ontario, Canada; 3 Department of Critical Care Medicine, Sunnybrook Health Sciences Centre, Toronto, Ontario, Canada; 4 Interdepartmental Division of Critical Care Medicine, University of Toronto, Toronto, Ontario, Canada; 5 Division of Infectious Diseases, Sunnybrook Health Sciences Centre and University of Toronto, Toronto, Ontario, Canada; 6 Division of Cardiology, Niagara Health and McMaster University, St. Catharines, Ontario, Canada; 7 Division of Cardiac Surgery, Sunnybrook Health Sciences Centre and University of Toronto, Toronto, Ontario, Canada; 8 Division of Cardiology, Sunnybrook Health Sciences Centre and University of Toronto, Toronto, Ontario, Canada; Ziekenhuisgroep Twente, NETHERLANDS

## Abstract

**Introduction:**

Accurate prediction of embolic events in infective endocarditis could inform critical clinical decisions, such as the timing of cardiac surgical intervention. However, many embolic events occur before hospital admission and echocardiography and are thus non-modifiable. We aimed to identify time-sensitive variables that predict embolic events in infective endocarditis, focusing on those that occur after diagnosis.

**Methods:**

Clinical, microbiological, and echocardiographic characteristics were collected from 116 patients with definite or probable left-sided infective endocarditis admitted to Sunnybrook Health Sciences Centre (Toronto, Canada) between October 2013 and July 2016; associations between these characteristics and embolic events were identified using simple logistic regression.

**Results:**

The mean (SD) age was 66 (17) years; 82 patients (71%) were men. The most frequent microorganisms were *Staphylococcus aureus* (23%) and viridans group streptococci (21%). Seventy-nine (68%) patients had left-sided vegetations, with involvement of the aortic valve in 34 (43%) patients, mitral valve in 37 (47%) patients, and both in 8 (10%) patients. The mean (SD) vegetation size was 10 (7) mm. Forty-three unique patients (37%) had 50 embolic events, with most (34/43; 79%) having a first embolic event (38/50; 76%) before or on the day of echocardiography. There were no significant predictors of the 11 patients with an embolic event after echocardiography; significant predictors of an embolic event at any time were single valve vegetation vs. no vegetation (OR, 4.75; 95% confidence interval [CI], 1.76–12.78) and, among patients with a vegetation, mitral vs. aortic valve location (OR, 4.43; 95%CI, 1.63–12.04).

**Conclusions:**

Associations between patient and echocardiographic characteristics and embolism in patients with infective endocarditis may be time-sensitive, as few embolic events occurred after clinical and echocardiographic assessment.

## Introduction

Infective endocarditis (IE) is characterized by high morbidity and mortality despite advances in medical and surgical care [1]. Among the most common and catastrophic complications of IE are embolic events (EEs). Echocardiographic variables associated with EEs include vegetation size [2–5], mobility [4, 6, 7], and mitral location [8–12]. Other clinical variables associated with EEs include *Staphylococcus aureus* infection [2, 6, 7, 9, 12–18], age [2, 18, 19], intravenous drug use (IVDU) [16, 20], and prosthetic valve IE [12, 17, 21].

Early surgery for IE patients at highest risk of EEs has been recommended in consensus guidelines [1, 22, 23]. However, many embolic complications of IE occur around the time of presentation and prior to echocardiography, reducing the clinical utility of EE prediction rules given that such EEs are not modifiable [3, 12, 15, 17, 21, 24, 25]. We therefore sought to identify variables predictive of EEs following the diagnosis of IE (primary objective), since these may be of the greatest utility to inform clinical decisions, including the timing of surgery.

## Materials and methods

### Study design and setting

We conducted a cohort study of patients with IE admitted to Sunnybrook Health Sciences Centre (SHSC), a tertiary care university-affiliated hospital in Toronto, Canada. We assessed the association between clinical, microbiological, and echocardiographic features and the risk of EEs; the primary analysis focused on new EEs occurring after echocardiography. The study was approved by the SHSC Research Ethics Board. The reporting of this study conforms to the STROBE statement [26] (Checklist in S1 Appendix).

### Patient identification

We included patients admitted to SHSC between 1 October 2013 and 1 July 2016 who met definite modified Duke criteria or probable criteria with strong clinical suspicion for active left-sided IE [27]. Patients admitted 1 October 2013 to 30 June 2015 were identified retrospectively by searching electronic discharge summaries for International Statistical Classification of Diseases and Related Health Problems (ICD-10-CM) codes I33 and I39, which have been validated for identification of patients with IE [28]. For patients with multiple encounters, the first was counted. Additional potential IE cases were captured by screening a hospital registry of heart valve surgeries and the microbiology database for blood cultures positive for microorganisms associated with IE (*Staphylococcus aureus*, *Streptococcus gallolyticus* (formerly *bovis*), HACEK organisms, *Enterococcus* spp., viridans group streptococci). Between 1 July 2015 and 1 July 2016, patients were identified prospectively by notification from the cardiac surgery, cardiology, or infectious diseases services, as part of a multi-disciplinary IE quality improvement program [29].

### Patient and pathogen characteristics

Patient characteristics were abstracted from electronic and paper medical records. Demographic factors included age, sex, and source of admission (direct vs. transfer from another hospital); comorbidities and risk factors included coronary artery disease, coronary artery bypass grafting, unrepaired valve lesion, prosthetic valve, intracardiac device, previous IE, intravenous drug use (IVDU), intravenous instrumentation (e.g., hemodialysis or central venous catheter), hypertension, diabetes mellitus, cerebrovascular disease, chronic kidney disease, liver disease, chronic obstructive pulmonary disease, and malignancy. We also recorded the timing of cardiac surgery for endocarditis, if performed. Microbiologic etiology was determined by microorganisms isolated from blood cultures performed using BACTEC 9240 system (Becton Dickinson Diagnostic Instruments Systems, USA), in accordance with Clinical Laboratory Standards Institute guidelines. Microorganisms were identified by VITEK 2 (model 510731-9EN1, bioMérieux Inc., USA).

### Echocardiographic characteristics

**Fig 1:**
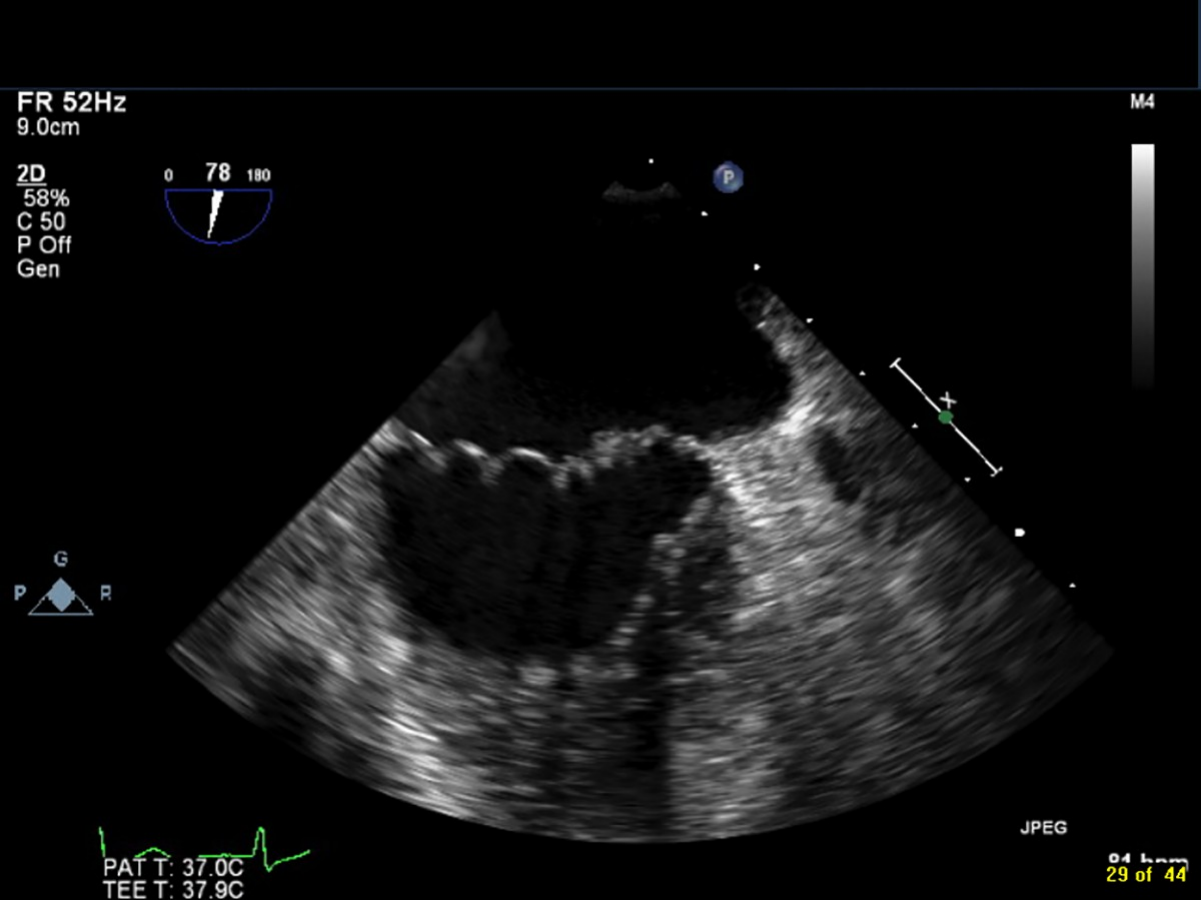
Example of a small fixed mitral valve vegetation (Grade 1 for both mobility and extent).

**Fig 2:**
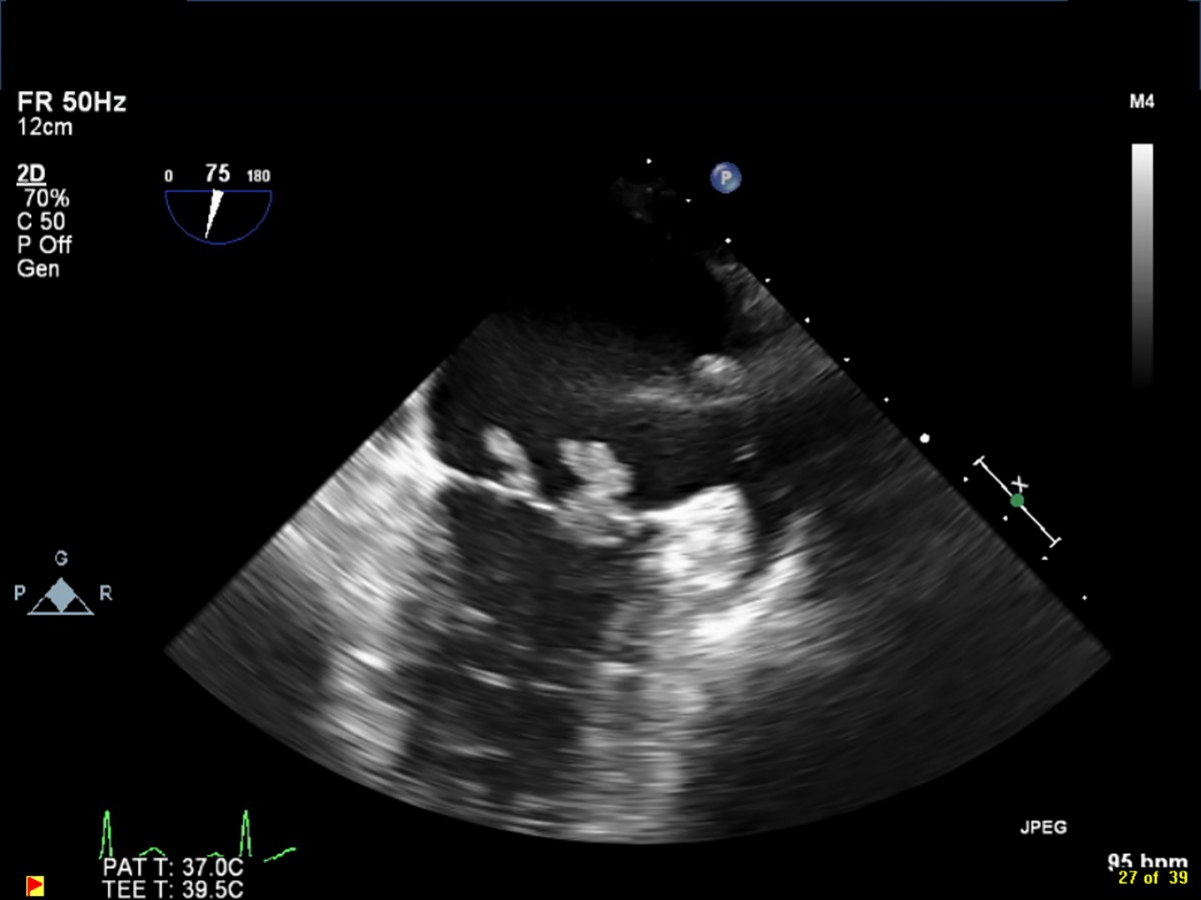
Example of large multiple mobile mitral valve vegetations (Grade 4 for mobility and Grade 2 for extent).

Transthoracic (2D) or transesophageal (2D or 3D) echocardiography was performed on all patients using Philips Medical IE33 equipment (Philips, Netherlands) and reviewed by two Level 3 certified cardiologists (BKMH and MSH); disagreements were resolved by consensus. Vegetations located on mitral or aortic valve leaflets were included, and right-sided vegetations were excluded from the analyses. Vegetation size was measured in multiple planes to determine the maximum length. In the case of multiple vegetations, the location and length of the largest vegetation was used. Mobility was described as follows: *Grade 1 (absent)*: fixed with no detectable independent motion; *Grade 2 (low)*: fixed base with mobile free edge; *Grade 3 (moderate)*: pedunculated vegetation that remains within the same chamber throughout the cardiac cycle; *Grade 4 (severe)*: prolapsing vegetations that cross the leaflet coaptation plane [18]. The extent of vegetation was categorized as follows: *Grade 1*: single vegetation; *Grade 2*: multiple vegetations limited to a single valve leaflet; *Grade 3*: involvement of multiple valve leaflets; *Grade 4*: vegetation that extends to extravalvular structures [18]. Fig 1 and S1 Video show a mitral valve vegetation that is Grade 1 in mobility and extent, and Fig 2 and S2 Video show multiple mitral valve vegetations on one leaflet (Grade 2 extent) that are Grade 4 in mobility.

### Outcomes

The primary outcome was the occurrence of an EE (stroke or major arterial embolic complication) determined clinically at least one day after echocardiography (referred to as ‘after echocardiography’) and within 6 weeks of admission. Patients with several EEs were counted as having experienced the primary outcome if one EE occurred in the above timing window. Minor embolic lesions (e.g. cutaneous lesions, splenic or renal infarcts) or clinically silent emboli were not considered. The secondary outcome included all EEs, regardless of timing.

### Statistical analysis

The number of patients with IE at SHSC during the study period determined the sample size. Continuous data are summarized as mean (standard deviation, SD) if normally distributed or median (first quartile, third quartile [Q1, Q3]) if not normally distributed. Categorical data are summarized as counts and percentages. There were no missing data for the exposures and outcomes of interest. Patients with >1 EE were counted once in the analysis, with the first EE analyzed, unless the subsequent EE occurred after echocardiography, in which case the subsequent EE is counted as the primary outcome. For echocardiographic characteristics, we used simple logistic regression to report the odds ratio (OR) and 95% confidence interval (CI) for the association of patient, pathogen, and echocardiographic characteristics with EEs. When any level of a factor had no EEs, we used exact logistic regression to handle the problem of quasi-complete separation that occurs with standard logistic regression. Multivariable analysis was not performed given a limited sample size. A *p* value <0.05 was considered statistically significant. All analyses were performed with Microsoft Excel (Microsoft Corporation, Redmond, WA, USA) and SAS version 9.4 (SAS, Cary, NC, USA).

## Results

### Patient and pathogen characteristics

A total of 116 patients were enrolled, including 80 patients (69%) with definite IE and 36 patients (31%) with probable IE. There were 82 men (71%) and 34 women (29%). The mean (SD) age was 66 (17) years. The most frequent comorbidities were hypertension (n = 61, 53%), prosthetic valve (n = 39, 34%), coronary artery disease (n = 34, 29%), malignancy (n = 29, 25%), cerebrovascular disease (n = 28, 24%), and diabetes mellitus (n = 27, 23%). The most frequent causative microorganisms were *Staphylococcus aureus* (n = 27, 23%), viridans group streptococci (n = 24, 21%), and *Enterococcus spp*. (n = 19, 16%). Additional clinical and microbiological characteristics are summarized in Table 1. Cardiac surgery was performed for 29 (25%) patients at a median (Q1, Q3) of 6 (3, 13) days after admission.

**Table 1:** Patient and pathogen characteristics of patients with or without an embolic event at any time.

Characteristic	Total Sample (n = 116)	Patients with EE (n = 43)	Patients without EE (n = 73)	Odds Ratio (95% CI)	*P* value
**Demographic characteristics**					
Mean age in years (SD)	66 (17)	63 (17)	67 (17)	0.99 (0.97–1.01)	0.25
Men	82 (70.7%)	30 (69.8%)	52 (71.2%)	0.93 (0.41–2.13)	0.87
Direct admission	71 (61.2%)	24 (55.8%)	47 (64.4%)	0.70 (0.32–1.51)	0.36
Definite Duke criteria	80 (69.0%)	39 (90.7%)	41 (56.2%)	7.61 (2.46–23.51)	<***0*.*001***
**Comorbidities and risk factors**					
Coronary artery disease	34 (29.3%)	11 (25.6%)	23 (31.5%)	0.75 (0.32–1.74)	0.50
Coronary artery bypass grafting	15 (12.9%)	5 (11.6%)	10 (13.7%)	0.83 (0.26–2.61)	0.75
Prosthetic valve(s)	39 (33.6%)	15 (34.9%)	24 (32.9%)	1.09 (0.49–2.42)	0.83
Unrepaired valve lesion	23 (19.8%)	9 (20.9%)	14 (19.2%)	1.12 (0.44–2.85)	0.82
Intracardiac device	13 (11.2%)	2 (4.7%)	11 (15.1%)	0.28 (0.06–1.31)	0.10
Prior infective endocarditis	12 (10.3%)	4 (9.3%)	8 (11.0%)	0.83 (0.24–2.95)	0.78
Intravenous drug use	7 (6.0%)	4 (9.3%)	3 (4.1%)	2.39 (0.51–11.24)	0.27
Intravenous instrumentation^a^	6 (5.2%)	1 (2.3%)	5 (6.8%)	0.32 (0.04–2.87)	0.31
Hypertension	61 (52.6%)	27 (62.8%)	34 (46.6%)	1.94(0.90–4.18)	0.093
Diabetes mellitus	27 (23.3%)	10 (23.3%)	17 (23.3%)	1.00 (0.41–2.44)	1
Cerebrovascular disease	28 (24.1%)	12 (27.9%)	16 (21.9%)	1.38 (0.58–3.28)	0.47
Chronic kidney disease	23 (19.8%)	5 (11.6%)	18 (24.7%)	0.40 (0.14–1.18)	0.089
Liver disease	12 (10.3%)	2 (4.7%)	10 (13.7%)	0.31 (0.06–1.48)	0.14
Chronic obstructive pulmonary disease	7 (6.0%)	3 (7.0%)	4 (5.5%)	1.29 (0.28–6.08)	0.74
Malignancy	29 (25.0%)	4 (9.3%)	25 (34.2%)	0.20 (0.06–0.61)	***0*.*0051***
Immunosuppression	9 (7.8%)	3 (7.0%)	6 (8.2%)	0.84 (0.20–3.54)	0.81
Obesity	7 (6.0%)	3 (7.0%)	4 (5.5%)	1.29 (0.28–6.08)	0.74
**Microbiologic etiology**					
*Staphylococcus aureus*	27 (23.3%)	11 (25.6%)	16 (21.9%)	1.23 (0.51–2.96)	0.65
Coagulase-negative staphylococcus	7 (6.0%)	2 (4.7%)	5 (6.9%)	0.66 (0.12–3.58)	0.63
*Enterococcus*	19 (16.4%)	3 (7.0%)	16 (21.9%)	0.27 (0.07–0.98)	***0*.*046***
Viridans group streptococcus	24 (20.7%)	10 (23.3%)	14 (19.2%)	1.28 (0.51–3.19)	0.60
HACEK group species^b^	2 (1.7%)	1 (2.3%)	1 (1.4%)	1.71 (0.10–28.13)	0.71
Other microorganism	32 (27.6%)	11 (25.6%)	21 (28.8%)	0.85 (0.36–2.00)	0.71
Culture-negative	8 (6.9%)	5 (11.6%)	3 (4.1%)	3.07 (0.70–13.55)	0.14

CI, confidence interval; EE, embolic event

^a^Hemodialysis or central venous catheter

^b^*Haemophilus*, *Aggregatibacter*, *Cardiobacterium*, *Eikenella*, and *Kingella* spp.

### Echocardiographic characteristics

Echocardiography was performed a median (Q1, Q3) of 3 (1, 5.5) days after admission, including one patient who was scanned at a referring hospital and admitted the following day to SHSC. Vegetations were detected in 79 patients (68%), of whom 37 (47%) had isolated mitral involvement, 34 (43%) had isolated aortic involvement, and 8 (10%) had bi-valvular involvement (Table 2). There were 63 patients (80%) with native valve involvement and 16 patients (20%) with prosthetic valve involvement. Vegetation size was ≥10 mm in 32 patients (40%) and <10 mm in 47 patients (60%), with a mean (SD) size of 10 (7) mm. Thirty-six patients (46%) had low (grades 1 or 2) vegetation mobility, while 43 patients (54%) had high (grades 3 or 4) mobility; 53 patients (67%) had low (grades 1 or 2) vegetation extent, while 26 patients (33%) had high (grades 3 or 4) extent. Thirty-seven patients (32%) had no vegetation detected (11/80 [14%] of patients with definite IE, 26/36 [72%] of patients with probable IE).

**Table 2:** Echocardiographic characteristics of patients with an embolic event occurring at any time.

Echocardiographic Characteristic	All patients (n = 116)	Patients with EE (n = 43)	Patients without EE (n = 73)	Odds Ratio (95% CI)	*P* value
**Number of valves with vegetation**					***0*.*0086***
None	37 (31.9%)	6 (14.0%)	31 (42.5%)	1	
Single	71 (61.2%)	34 (79.1%)	37 (50.7%)	4.75 (1.76–12.78)	***0*.*0021***
Bi-valvular	8 (6.9%)	3 (7.0%)	5 (7.0%)	3.10 (0.58–16.59)	0.19
	**Patients with valves with vegetation** **(n = 79)**	**(n = 37)**	**(n = 42)**		
**Valve type**					
Native	63 (79.8%)	26 (70.3%)	37 (88.1%)	1	
Prosthetic	16 (20.3%)	11 (29.7%)	5 (11.9%)	3.13 (0.97–10.09)	0.056
**Valve location**					***0*.*012***
Aortic	34 (43.0%)	10 (27.03%)	24 (57.1%)	1	
Mitral	37 (46.8%)	24 (64.9%)	13 (31.0%)	4.43 (1.63–12.04)	***0*.*0035***
Bivalvular	8 (10.1%)	3 (8.1%)	5 (11.9%)	1.44 (0.29–7.21)	0.66
**Vegetation size**					
Size <10 mm	47 (59.5%)	20 (54.1%)	27 (64.3%)	1	
Size ≥10 mm	32 (40.5%)	17 (46.0%)	15 (35.7%)	1.53 (0.62–3.78)	0.36
Size (mean, in mm)	10.4 (6.6)	11.6 (7.6)	9.3 (5.4)	1.06 (0.98–1.13)^a^	0.13
**Vegetation mobility**					0.81
Grade 1	6 (7.6%)	3 (8.1%)	3 (7.1%)	1	
Grade 2	30 (38.0%)	12 (32.4%)	18 (42.9%)	0.67 (0.12–3.87)	0.65
Grade 3	13 (16.5%)	7 (18.9%)	6 (14.3%)	1.17 (0.17–8.09)	0.88
Grade 4	30 (38.0%)	15 (40.5%)	15 (35.7%)	1.00 (0.17–5.77)	1.00
**Vegetation extent**					0.38
Grade 1	32 (40.5%)	12 (32.4%)	20 (47.6%)	1	
Grade 2	21 (26.6%)	13 (35.1%)	8 (19.1%)	2.71 (0.87–8.43)	0.09
Grade 3	21 (26.6%)	10 (27.0%)	11 (26.2%)	1.52 (0.50–4.63)	0.47
Grade 4	5 (6.3%)	2 (5.4%)	3 (7.1%)	1.11 (0.16–7.63)	0.91

CI, confidence interval; EE, embolic event

^a^Odds Ratio per 1-mm increase in vegetation size.

### Embolic events (EEs)

**Fig 3:**
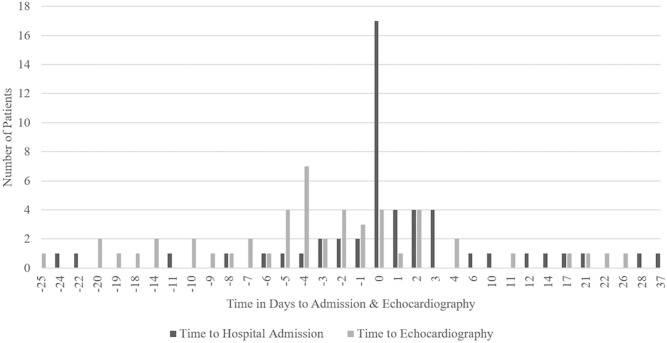
Timing of EEs in relation to hospital admission and echocardiography. Most EEs occurred before or on the day of admission or echocardiography. EE, embolic event.

Forty-three patients (37%) had EEs, with the majority experiencing the first EE before or on the day of hospital admission (30/43, 70%) and before or on the day of echocardiography (34/43, 79%). Seven (16%) patients experienced 2 EEs, of whom 4 (9%) had both EEs before or on the day of echocardiography, 2 (5%) had an EE both before and after echocardiography, and 1 (2%) had both EEs after echocardiography. Eleven patients met the primary outcome definition of having an EE after echocardiography, including 2 patients who also had an EE before echocardiography. The clinical course of patients with the primary outcome is summarized in Table A in S1 Appendix; in 10 of 11 patients an EE was a stroke. In total, there were 50 EEs, including 47 (94%) strokes, 2 (4%) ischemic limb events, and 1 (2%) ischemic bowel event. Twelve (24%), 18 (36%), and 20 (40%) EEs occurred before, on the day of, and after admission, respectively. Thirty-four (68%), 4 (8%), and 12 (24%) EEs occurred before, on the day of, and after echocardiography, respectively (Fig 3).

### Clinical and microbiological variables associated with EEs

As presented in Table 1, patients who met definite Duke criteria had a higher risk of any EE (before or after hospital admission) than those who only met probable Duke criteria (odds ratio [OR] 7.61; 95%CI, 2.46–23.51; *p*<0.001). Decreased probability of any EE was associated with malignancy (OR 0.20; 95%CI, 0.06–0.61; *p* = 0.0051) and *Enterococcus* spp. infection (OR 0.27; 95%CI, 0.07–0.98; *p* = 0.046). There were no associations with any other comorbidities or causative microorganisms.

There were no significant differences in clinical or microbiological characteristics between patients with and without an EE occurring after echocardiography (Table B in S1 Appendix).

### Echocardiographic variables predictive of EEs after echocardiography

No factors were associated with a statistically significantly higher risk of subsequent EEs (Table 3), including single valve vegetation (OR 2.21; 95%CI, 0.48–14.06; *p* = 0.42), mitral valve vegetation (OR 3.00; 95%CI, 0.49–22.46; *p* = 0.30), and vegetation size ≥10 mm (OR 1.80; 95%CI, 0.39–8.25; *p* = 0.45), mobility (*p* = 0.92), and extent (*p* = 0.39).

**Table 3:** Echocardiographic characteristics of patients with an embolic event occurring after echocardiography.

Echocardiographic Characteristic	All patients (n = 84)^a^	Patients with EE (n = 11)^b^	Patients without EE (n = 73)	Odds Ratio (95% CI)	*P* value
**Number of valves with vegetation**					0.41
None	34 (40.5%)	3 (27.3%)	31 (42.5%)	1	
Single	45 (53.6%)	8 (72.7%)	37 (50.7%)	2.21 (0.48–14.06)	0.42
Bi-valvular	5 (6.0%)	0 (0%)	5 (6.9%)	1.75 (0–12.87)	0.65
	**Patients with valves with vegetation** **(n = 50)**	**(n = 8)**	**(n = 42)**		
**Valve type**					
Native	43 (86.0%)	6 (75.0%)	37 (88.1%)	1	
Prosthetic	7 (14.0%)	2 (25.0%)	5 (11.9%)	2.47 (0.39–15.73)	0.34
**Valve location**					0.26
Aortic	27 (54.0%)	3 (37.5%)	24 (57.1%)	1	
Mitral	18 (36.0%)	5 (62.5%)	13 (31.0%)	3.00 (0.49–22.46)	0.30
Bivalvular	5 (10.0%)	0 (0%)	5 (11.9%)	1.37 (0–10.10)	0.59
**Vegetation size**					
Size <10 mm	31 (62.0%)	4 (50.0%)	27 (64.3%)	1	
Size ≥10 mm	19 (38.0%)	4 (50.0%)	15 (35.7%)	1.80 (0.39–8.25)	0.45
Size (mean, in mm)	9.7 (5.5)	11.8 (5.9)	9.3 (5.4)	1.08 (0.95–1.23)^c^	0.25
**Vegetation mobility**					0.92
Grade 1	3 (6.0%)	0 (0%)	3 (7.1%)	1	
Grade 2	21 (42.0%)	3 (37.5%)	18 (42.9%)	0.56 (0.07-∞)	0.66
Grade 3	7 (14.0%)	1 (12.5%)	6 (14.3%)	0.43 (0.02-∞)	0.70
Grade 4	19 (38.0%)	4 (50.0%)	15 (35.7%)	0.90 (0.12-∞)	0.53
**Vegetation extent**					0.39
Grade 1	22 (44.0%)	2 (25.0%)	20 (47.6%)	1	
Grade 2	9 (18.0%)	1 (12.5%)	8 (19.1%)	1.25 (0.10–15.80)	0.86
Grade 3	14 (28.0%)	3 (37.5%)	11 (26.2%)	2.73 (0.39–18.88)	0.31
Grade 4	5 (10.0%)	2 (25.0%)	3 (7.1%)	6.67 (0.67–66.84)	0.11

CI, confidence interval; EE, embolic event

^a^The total sample excludes patients who had EEs exclusively before echocardiography.

^b^Of 11 patients with EEs, 8 had vegetations: 5 had a single EE after echocardiography, 1 had two EEs after echocardiography, and 2 had one EE before and one EE after echocardiography. The 3 patients without vegetations each had 1 EE after echocardiography.

^c^Odds Ratio per 1-mm increase in vegetation size.

### Echocardiographic variables associated with EEs at any time

Among all patients, single valve vs. no vegetation (OR 4.75; 95%CI, 1.76–12.78; *p* = 0.0021) was associated with any EE. Among patients with a vegetation, mitral vs. aortic valve location (OR 4.43; 95%CI, 1.63–12.04; *p* = 0.0035) was associated with higher risk of any EE. Vegetation size ≥10 mm (OR, 1.53; 95%CI, 0.62–3.78; *p* = 0.36), mobility (*p* = 0.81), and extent (*p* = 0.38) demonstrated no statistically significant association (Table 2).

## Discussion

We investigated 116 patients with definite or probable IE to identify clinical, microbiological, and echocardiographic characteristics associated with EEs. EEs occurred in 37% of patients, congruent with previously reported rates of 18–44% [17, 24, 30, 31]. The large majority of EEs occurred before or on the day of hospital admission, thereby precipitating medical contact. We found several predictors of all EEs that were no longer statistically significant when considering only EEs that occurred after echocardiography, limiting their utility for clinical decision-making.

Echocardiographic variables predictive of EEs included single valve vegetation [3, 32] and mitral valve vegetation [8, 10–12], congruent with published findings; however, we observed that the relationship between the same variables and EEs subsequent to echocardiography was attenuated. Although this is likely a consequence of insufficient statistical power, an attenuated relationship between EEs after echocardiography and vegetation presence and location has been reported in larger sample sizes [4, 31]. The relationship between echocardiographic variables and subsequent EEs is likely to be complex and time-dependent; the apparent cause-and-effect of vegetation characteristics and embolization depends on when they are visualized. In our study, vegetation size ≥10 mm was not significantly associated with any EEs or only those occurring after echocardiography. However, a recent meta-analysis of 7 studies (3253 patients) of left-sided IE found vegetation size >10mm to be associated with any EEs but not with new EEs occurring after admission, echocardiography, or antibiotic therapy initiation [33]. Another meta-analysis of 21 studies (6646 patients) including both left- and right-sided IE found native valve vegetation size >10 mm to be significantly associated with increased odds of any EE, but did not examine the relationship with post-admission or post-echocardiography EEs [34].

We found that increased rates of any EE were associated with definite IE according to Duke criteria [30, 35] and absence of malignancy [17], but not with coagulase-negative staphylococcal or viridans group streptococcal infections [14, 17, 24], in keeping with prior reports. We did not observe an association between EEs and *Staphylococcus aureus* infection despite previous observations [6, 12–17]. Cancer patients with indwelling vascular devices and probable but not definite endocarditis may have confounded this association.

A randomized controlled trial of early surgery for mobile left-sided vegetations demonstrated reductions in EEs, and consideration of early surgery has been incorporated into practice guidelines [36]. The present findings emphasize that the majority of EEs occur prior to or at the time of admission; subsequent events are infrequent, in keeping with previous reports suggesting that embolism is uncommon after initiation of antibiotics [2].

This study has several limitations, which may limit its generalizability. As the study was conducted in a single tertiary care university-affiliated hospital that receives transfers from other institutions, the findings may be subject to referral bias. Retrospective data may be subject to under-detection of outcomes due to unreported complications or incomplete chart documentation, limitations less applicable to the phase of the study with prospective patient identification. The small number of EEs after echocardiography limited statistical power. Inclusion of a high proportion of probable IE patients (31% compared to 7–11% seen in large multicentre studies [9, 17, 30]), of which a majority had no echocardiographic findings, may have also diluted the observed associations. Finally, the performance of cardiac surgery may have reduced the risk of a subsequent EE.

## Conclusions

The present study validates a global association between conventional clinical and echocardiographic variables and systemic embolism, while suggesting that the predictive value of echocardiographic variables may be time-sensitive. These results illustrate limitations inherent in studies that combine prospective and retrospective associations with embolism and serve as a caveat emphasizing the importance of a comprehensive evaluation when applying practice guidelines in planning surgical intervention.

## Supporting information

S1 Appendix Additional information about the study. The file consists of (1) Strengthening the Reporting of Observational studies in Epidemiology (STROBE) checklist; (2) Table A: Clinical course of patients with an embolic event occurring after echocardiography; and (3) Table B: Patient and pathogen characteristics of patients with or without the primary outcome of an embolic event after echocardiography.
(PDF)

S1 Video Example of a small fixed mitral valve vegetation (Grade 1 for both mobility and extent). (AVI)

S2 Video Example of large multiple mobile mitral valve vegetations (Grade 4 for mobility and Grade 2 for extent). (AVI)
