# Supplementary material for: Time-sensitive predictors of embolism in patients with left-sided endocarditis: Cohort study
Source: PLoS One. 2019 Apr 25;14(4):e0215924. doi: 10.1371/journal.pone.0215924 (PMC6483226; doi:10.1371/journal.pone.0215924)
Supplement: S1 Appendix — The file consists of (1) Strengthening the Reporting of Observational studies in Epidemiology (STROBE) checklist; (2) Table A: Clinical course of patients with an embolic event occurring after echocardiography; and (3) Table B: Patient and pathogen characteristics of patients with or without the primary outcome of an embolic event after echocardiography. (PDF) [file pone.0215924.s001.pdf]

**S1 Appendix**

**Strengthening the Reporting of Observational studies in Epidemiology (STROBE) checklist** of items that should be included in reports of *cohort studies*

| | **Item No** | **Recommendation** |
|---|---|---|
| **Title and abstract** | 1 | (*a*) Indicate the study's design with a commonly used term in the title or the abstract **(p. 1)** |
| | | (*b*) Provide in the abstract an informative and balanced summary of what was done and what was found **(p. 2)** |
| **Introduction** | | |
| Background/rationale | 2 | Explain the scientific background and rationale for the investigation being reported **(p. 3)** |
| Objectives | 3 | State specific objectives, including any prespecified hypotheses **(p. 3)** |
| **Methods** | | |
| Study design | 4 | Present key elements of study design early in the paper **(p. 3)** |
| Setting | 5 | Describe the setting, locations, and relevant dates, including periods of recruitment, exposure, follow-up, and data collection **(pp. 3-4)** |
| Participants | 6 | (*a*) Give the eligibility criteria, and the sources and methods of selection of participants. Describe methods of follow-up **(p. 4)** |
| | | (*b*) For matched studies, give matching criteria and number of exposed and unexposed **(not applicable)** |
| Variables | 7 | Clearly define all outcomes, exposures, predictors, potential confounders, and effect modifiers. Give diagnostic criteria, if applicable **(pp. 4-5)** |
| Data sources/ measurement | 8* | For each variable of interest, give sources of data and details of methods of assessment (measurement). Describe comparability of assessment methods if there is more than one group **(pp. 4-5)** |
| Bias | 9 | Describe any efforts to address potential sources of bias **(pp.4- 5)** |
| Study size | 10 | Explain how the study size was arrived at **(p. 6)** |
| Quantitative variables | 11 | Explain how quantitative variables were handled in the analyses. If applicable, describe which groupings were chosen and why **(p. 6)** |
| Statistical methods | 12 | (*a*) Describe all statistical methods, including those used to control for confounding **(pp. 6-7)** |
| | | (*b*) Describe any methods used to examine subgroups and interactions **(not applicable)** |
| | | (*c*) Explain how missing data were addressed **(not applicable)** |
| | | (*d*) If applicable, explain how loss to follow-up was addressed **(not applicable)** |
| | | (*e*) Describe any sensitivity analyses **(not applicable)** |
| **Results** | | |
| Participants | 13* | (a) Report numbers of individuals at each stage of study—eg numbers potentially eligible, examined for eligibility, confirmed eligible, included in the study, completing follow-up, and analysed **(p. 7)** |
| | | (b) Give reasons for non-participation at each stage **(not applicable)** |
| | | (c) Consider use of a flow diagram **(not applicable)** |

| | | |
|---|---|---|
| Descriptive data | 14* | (a) Give characteristics of study participants (eg demographic, clinical, social) and information on exposures and potential confounders **(p. 7, Table 1)** |
| | | (b) Indicate number of participants with missing data for each variable of interest **(not applicable)** |
| | | (c) Summarise follow-up time (eg, average and total amount) **(not applicable)** |
| Outcome data | 15* | Report numbers of outcome events or summary measures over time **(Table 1, Table 2, Table 3)** |
| Main results | 16 | (*a*) Give unadjusted estimates and, if applicable, confounder-adjusted estimates and their precision (eg, 95% confidence interval). Make clear which confounders were adjusted for and why they were included **(p. 11, p. 13)** |
| | | (*b*) Report category boundaries when continuous variables were categorized **(Table 2, Table 3)** |
| | | (*c*) If relevant, consider translating estimates of relative risk into absolute risk for a meaningful time period **(not applicable)** |
| Other analyses | 17 | Report other analyses done—eg analyses of subgroups and interactions, and sensitivity analyses **(not applicable)** |
| **Discussion** | | |
| Key results | 18 | Summarise key results with reference to study objectives **(pp. 13-14)** |
| Limitations | 19 | Discuss limitations of the study, taking into account sources of potential bias or imprecision. Discuss both direction and magnitude of any potential bias **(pp. 14-15)** |
| Interpretation | 20 | Give a cautious overall interpretation of results considering objectives, limitations, multiplicity of analyses, results from similar studies, and other relevant evidence **(pp. 13-15)** |
| Generalisability | 21 | Discuss the generalisability (external validity) of the study results **(p. 14)** |
| **Other information** | | |
| Funding | 22 | Give the source of funding and the role of the funders for the present study and, if applicable, for the original study on which the present article is based **(see separate funding statement)** |

*Information given separately for exposed and unexposed groups.
Note that page numbers refer to the Microsoft Word version of the revised manuscript.

**Table A. Clinical course of patients with an embolic event occurring after echocardiography.**

| Patient # (age and sex) | Echocardiography | | Embolic Event | Management | Vital status at hospital discharge |
|---|---|---|---|---|---|
| | Days after admission performed | Vegetation description | | | |
| 1 (42M) | 0 | Single 9 mm vegetation on prosthetic aortic valve | 1 non-stroke EE occurring 21 days after echocardiography | Antibiotic therapy + aortic valve replacement 19 days after echocardiography | Dead |
| 2 (46M) | 1 | Single 6 mm vegetation on native mitral valve | 1 stroke occurring 1 day after echocardiography | Antibiotic therapy | Alive |
| 3 (51M) | 2 | None | 1 stroke occurring 4 days after echocardiography | Antibiotic therapy | Alive |
| 4 (67M) | 15 | Single 17 mm vegetation on native aortic valve | 2 strokes occurring 20 days before and 22 days after echocardiography | Antibiotic therapy | Alive |
| 5 (86M) | 2 | None | 1 stroke occurring 26 days after echocardiography | Antibiotic therapy | Alive |
| 6 (79F) | 10 | Single 5 mm vegetation prosthetic mitral valve | 1 stroke occurring 4 days after echocardiography | Antibiotic therapy | Dead |
| 7 (42M) | 1 | Single 15 mm vegetation on native aortic valve | 2 strokes occurring 1 day before and 11 days after echocardiography | Antibiotic therapy | Alive |
| 8 (44M) | 0 | Single 20 mm vegetation on native mitral valve | 1 non-stroke EE and 1 stroke occurring 2 and 17 days after echocardiography respectively | Antibiotic therapy + mitral valve replacement 1 day after echocardiography | Alive |
| 9 (86F) | 1 | None | 1 stroke occurring 2 days after echocardiography | Antibiotic therapy | Alive |
| 10 (41M) | 0 | Single 16 mm vegetation on native mitral valve | 1 stroke occurring 2 days after echocardiography | Antibiotic therapy + mitral valve replacement 5 days after echocardiography | Alive |
| 11 (75F) | 1 | Single 6 mm vegetation on native mitral valve | 1 stroke occurring 2 days after echocardiography | Antibiotic therapy | Alive |

EE, embolic event; M, male; F, female

**Table B. Patient and pathogen characteristics of patients with or without the primary outcome of an embolic event after echocardiography.**

| Characteristic | Total Population (n=116) | Patients with primary outcome (n=11) | Patients without primary outcome (n=105) | Odds Ratio (95% CI) | *P* value |
|---|---|---|---|---|---|
| **Demographic characteristics** | | | | | |
| Mean age in years (SD) | 66 (17) | 60 (19) | 67 (17) | 0.98 (0.95-1.01) | 0.23 |
| Men | 82 (70.7%) | 8 (72.7%) | 74 (70.5%) | 1.12 (0.28-4.49) | 0.88 |
| Direct admission | 71 (61.2%) | 5 (45.5%) | 66 (62.9%) | 0.49 (0.14-1.72) | 0.27 |
| Definite Duke Criteria | 80 (69.0%) | 9 (81.8%) | 71 (67.6%) | 2.16 (0.44-10.52) | 0.34 |
| **Comorbidities and risk factors** | | | | | |
| Coronary artery disease | 34 (29.3%) | 3 (27.3%) | 31 (29.5%) | 0.90 (0.22-3.60) | 0.88 |
| Coronary artery bypass grafting | 15 (12.9%) | 0 (0%) | 15 (14.3%) | 0.40 (0-2.09) | 0.20 |
| Prosthetic valve(s) | 39 (33.6%) | 3 (27.3%) | 36 (34.3%) | 0.72 (0.18-2.88) | 0.64 |
| Unrepaired valve lesion | 23 (19.8%) | 2 (18.2%) | 21 (20.0%) | 0.89 (0.18-4.43) | 0.89 |
| Intracardiac device | 13 (11.2%) | 0 (0%) | 13 (12.4%) | 0.48 (0-2.49) | 0.25 |
| Prior infective endocarditis | 12 (10.3%) | 1 (9.1%) | 11 (10.5%) | 0.86 (0.10-7.33) | 0.89 |
| Intravenous drug use | 7 (6.0%) | 1 (9.1%) | 6 (5.7%) | 1.65 (0.18-15.11) | 0.66 |
| Intravenous instrumentation [a] | 6 (5.2%) | 1 (9.1%) | 5 (4.8%) | 2.00 (0.21-18.85) | 0.54 |
| Hypertension | 61 (52.6%) | 6 (54.6%) | 55 (52.4%) | 1.09 (0.31-3.80) | 0.89 |
| Diabetes mellitus | 27 (23.3%) | 2 (18.2%) | 25 (23.8%) | 0.71 (0.14-3.51) | 0.68 |
| Cerebrovascular disease | 28 (24.1%) | 2 (18.2%) | 26 (24.8%) | 0.68 (0.14-3.33) | 0.63 |
| Chronic kidney disease | 23 (19.8%) | 2 (18.2%) | 21 (20.0%) | 0.89 (0.18-4.43) | 0.89 |
| Liver disease | 12 (10.3%) | 0 (0%) | 12 (11.4%) | 0.52 (0-2.75) | 0.28 |
| Chronic obstructive pulmonary disease | 7 (6.0%) | 1 (9.1%) | 6 (5.7%) | 1.65 (0.18-15.11) | 0.66 |
| Malignancy | 29 (25.0%) | 1 (9.1%) | 28 (26.7%) | 0.28 (0.03-2.25) | 0.23 |
| Immunosuppression | 9 (7.8%) | 1 (9.1%) | 8 (7.6%) | 1.21 (0.14-10.71) | 0.86 |
| Obesity | 7 (6.0%) | 1 (9.1%) | 6 (5.7%) | 1.65 (0.18-15.11) | 0.66 |
| **Microbiologic etiology** | | | | | |
| *Staphylococcus aureus* | 27 (23.3%) | 4 (36.4%) | 23 (21.9%) | 2.04 (0.55-7.57) | 0.29 |
| Coagulase-negative staphylococcus | 7 (6.0%) | 0 (0%) | 7 (6.7%) | 0.96 (0-5.36) | 0.49 |
| *Enterococcus* | 19 (16.4%) | 0 (0%) | 19 (18.1%) | 0.30 (0-1.55) | 0.13 |
| Viridans group streptococcus | 24 (20.7%) | 3 (27.3%) | 21 (20.0%) | 1.50 (0.37-6.15) | 0.57 |
| HACEK group species [b] | 2 (1.7%) | 0 (0%) | 2 (1.9%) | 3.98 (0-34.18) | 0.82 |
| Other microorganism | 32 (27.6%) | 3 (27.3%) | 29 (27.6%) | 0.98 (0.24-3.96) | 0.98 |
| Culture-negative | 8 (6.9%) | 1 (9.1%) | 7 (6.7%) | 1.40 (0.16-12.56) | 0.76 |

[a] Hemodialysis or central venous catheter
[b] *Haemophilus, Aggregatibacter, Cardiobacterium, Eikenella,* and *Kingella* spp.
